# Anisotropic shear stress patterns predict the orientation of convergent tissue movements in the embryonic heart

**DOI:** 10.1242/dev.152124

**Published:** 2017-12-01

**Authors:** Francesco Boselli, Emily Steed, Jonathan B. Freund, Julien Vermot

**Affiliations:** 1 Institut de Génétique et de Biologie Moléculaire et Cellulaire, 67404 Illkirch, France; 2 Centre National de la Recherche Scientifique, UMR7104, 67404 Illkirch, France; 3 Institut National de la Santé et de la Recherche Médicale, U964, 67404 Illkirch, France; 4 Mechanical Science & Engineering, University of Illinois at Urbana-Champaign, Urbana, IL 61801, USA

**Keywords:** Fluid mechanics, Low Reynolds number, Red blood cells, *Danio rerio*, Morphogenesis, Photoconversion, Live imaging

## Abstract

Myocardial contractility and blood flow provide essential mechanical cues for the morphogenesis of the heart. In general, endothelial cells change their migratory behavior in response to shear stress patterns, according to flow directionality. Here, we assessed the impact of shear stress patterns and flow directionality on the behavior of endocardial cells, the specialized endothelial cells of the heart. At the early stages of zebrafish heart valve formation, we show that endocardial cells are converging to the valve-forming area and that this behavior depends upon mechanical forces. Quantitative live imaging and mathematical modeling allow us to correlate this tissue convergence with the underlying flow forces. We predict that tissue convergence is associated with the direction of the mean wall shear stress and of the gradient of harmonic phase-averaged shear stresses, which surprisingly do not match the overall direction of the flow. This contrasts with the usual role of flow directionality in vascular development and suggests that the full spatial and temporal complexity of the wall shear stress should be taken into account when studying endothelial cell responses to flow *in vivo*.

## Abstract (precis)

**Summary:** Blood flow modeling shows that dynamic shear stress patterns, rather than mean flow direction, predict the stereotypical behavior of endocardial cells during the early steps of heart valve formation.

## INTRODUCTION

Shortly after the first heartbeat, a directional blood flow is established in the embryonic cardiovascular system. The resulting flow forces trigger specific endocardial cell responses that are essential to the orchestration of the morphogenetic events leading to heart development, including chamber ballooning, valve formation and trabeculation (Boselli et al., 2015; Collins and Stainier, 2016; Haack and Abdelilah-Seyfried, 2016). The tangential force generated by fluid flow at the cell surface can be characterized in terms of shear stress (Freund et al., 2012). The importance of shear stress and its temporal profile has been extensively discussed. For example, oscillatory shear stress has been shown to modulate the expression of *klf2a*, an important flow-responsive gene for heart chamber (Dietrich et al., 2014) and valve development (Vermot et al., 2009; Heckel et al., 2015; Steed et al., 2016a). It is not clear, however, how the pattern defined by spatial changes of shear stress and its dynamics could potentially impact endocardial cell behavior. A major role might be anticipated because microcirculation flow polarizes endothelial cells and orients their migration according to shear stress spatial gradients, with motion opposite to flow direction, as observed *in vitro* (Ostrowski et al., 2014) and *in vivo*, during vascular morphogenesis (Kochhan et al., 2013; Franco et al., 2015; Kwon et al., 2016).

To identify the morphogenetic response of endocardial cells (EdCs) to shear stress patterns *in vivo*, we examined the early events of the formation of the atrioventricular canal (AVC), which is the area between the forming atrium and ventricle, where the valve will develop and where strong local spatial and temporal gradients are expected.

## RESULTS AND DISCUSSION

### EdCs converge toward the AVC at the onset of valvulogenesis

**Fig. 1.**
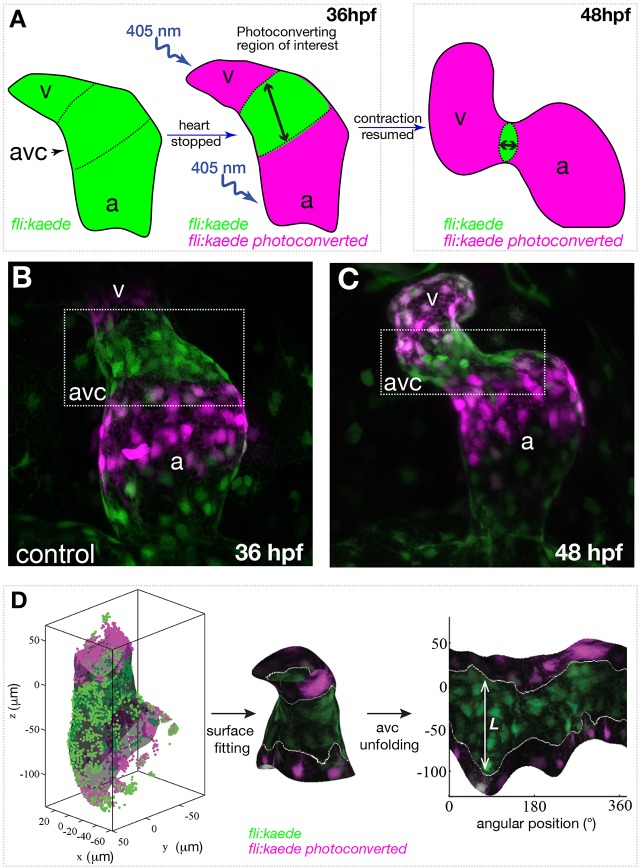
**Schematic of photoconversion experiments and of the three-dimensional analysis of early AVC morphogenesis.** (A) The atrium (a) and ventricle (v) of a 36 hpf *fli:kaede* heart are exposed to 405 nm UV light to photoconvert the genetically encoded Kaede protein from its green to red configuration. The same heart is imaged at 36 hpf and at 48 hpf to assay the movement of the photoconverted tissue. (B,C) Maximum intensity projection of a *fli:kaede* heart at 36 hpf and 48 hpf, respectively. (D) AVC segmentation and analysis: the endocardium of the AVC region is segmented with a parametric surface. The color intensity of the 3D dataset is projected on the parametric surface and the AVC is unfolded for 2D visualization and quantification of the length *L* of the photoconverted tissue.

**Fig. 2.**
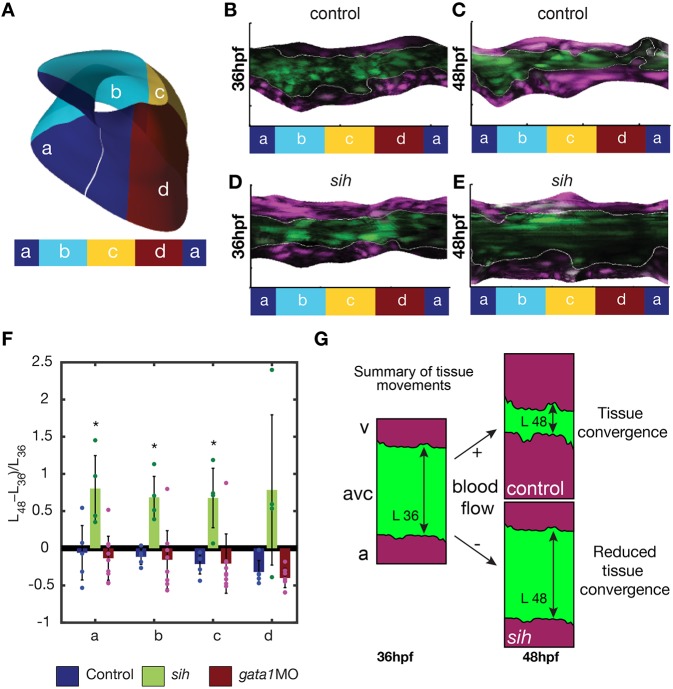
**Tissue convergence** **(AVC shortening) is flow dependent.** (A) Schematic showing the compartmentalization into superior (a), inferior (c), exterior (b) and interior (d) regions of the AVC. (B-E) Unfolded visualization of the *fli:kaede* (control) (B,C) and *sih;fli:kaede* AVC endocardium (D,E) at 36 hpf (B,D) and 48 hpf (C,E). (F) The shortening index (*L*_48_−*L*_36_)/*L*_36_ of the AVC between 36 hpf and 48 hpf in control (*n*=5), *sih* (*n*=4) and *gata1* (*n*=9) morphants is averaged on the a,b,c,d regions. *L*_48_ and *L*_36_ are the AVC length *L* at 48 hpf and 36 hpf, respectively. Data are mean±s.d. Statistical significance was determined by unpaired Student's *t*-test; *0.01<*P*<0.05. (G) Schematic of flow-dependent tissue convergence.

In recent studies of the morphogenetic events leading to valve emanation, the number of EdCs in the AVC has been shown to increase significantly between 36 hpf and 48 hpf (Pestel et al., 2016; Steed et al., 2016b). To characterize the corresponding morphogenetic events, we took advantage of the *Tg(fli:kaede)* line, which expresses Kaede, a photosensitive protein that changes its emission spectrum after exposure to UV light (Ando et al., 2002), and is of great use to study cardiovascular morphogenesis in zebrafish (Chow and Vermot, 2017). We photoconverted the Kaede in the atrium and ventricle of hearts at 36 hpf (Fig. 1A) and imaged them at 36 hpf and 48 hpf (see Materials and Methods) (Fig. 1B,C). To capture the full three-dimensional process, we developed an image analysis approach based on segmentation and unfolding of the AVC region (Fig. 1D and Fig. 2; Materials and Methods), facilitated by the endocardium being a monolayer at this stage. In short, a reference system is introduced that defines each point on the AVC by its axial position along the AVC centerline and its azimuthal angle around that axis. The endocardium is then segmented on this parametric surface, with the image intensity projected onto it. This yields a two-dimensional image with each column and row corresponding to a well-defined azimuthal and arc-length position, respectively. The edges between the photoconverted and nonphotoconverted cells are then easily found (Fig. 1D; Materials and Methods). We measure the AVC lengths *L*_36_ and *L*_48_ at 36 hpf and 48 hpf, respectively, and compute the AVC shortening factor (*L*_48_−*L*_36_)/*L*_36_ as a function of the angular position. The shortening factor is averaged over the superior, inferior, interior and exterior regions of the AVC (Fig. 2A), which are defined based on the orientation of the elliptic cross-section of the AVC and its orientation within the embryo. For the wild type (Fig. 2B,C), we found that the distance between the photoconverted areas decreases in size and that the average AVC shortening factor varies from about −0.3 to −0.06 (Fig. 2F). Considering that these cells will contribute to the AVC valve structure (Steed et al., 2016b; Pestel et al., 2016), these results suggest that the convergence of EdCs toward the AVC might be the first morphogenetic step of heart valve morphogenesis.

### EdC convergence toward the AVC is dependent upon mechanical forces

Early myocardial function has been shown to affect AVC development (Bartman et al., 2004). We thus assessed if this observed tissue movement is dependent on mechanical forces. Exploiting the well-documented ability of the zebrafish embryos to survive and develop without a functioning cardiovascular system until ∼5 dpf (Stainier et al., 1996), we first repeated the photoconversion experiment in *silent heart* (*sih*; *tnnt2a* – ZFIN) mutants, which lack heart contraction (Sehnert et al., 2002). Strikingly, the AVC shortening factor became positive, varying from ∼0.7 to 0.8 (Fig. 2D-F), indicating that the two photoconverted regions are in fact moving apart (Fig. 2F). We found in particular that the superior, inferior and exterior AVC shortening was different from the control (*P*<0.05). This suggests that cells require particular mechanical forces to cue convergence toward the center of the AVC (Fig. 2G).

### Red blood cells are not required for tissue convergence

As red blood cells (RBCs) have been suggested to provide mechanical cues to endothelial cells (Freund and Vermot, 2014), we next tested the influence of RBCs during tissue convergence. For this we studied *gata1* (*gata1a*) morphants, which almost totally inhibits hematopoiesis (Galloway et al., 2005). In these embryos, heart contractility is similar to that of controls (Vermot et al., 2009). We found that the AVC shortening factor varies from about −0.4 to −0.13 and was not significantly different from that of the controls, though with increased variability between embryos (Fig. 2F). Overall, these results suggest that RBCs are not essential to drive EdC convergence in the AVC, though this does not preclude some role in fine tuning the process.

### Fluid profiles in the AVC

The observed influence of mechanical forces on tissue convergence and the absence of a role for RBCs led us to assess the hemodynamics in more detail, in particular the directionality of the flow, generated between 33 hpf and 48 hpf. At these developmental stages, the beating heart works as a valveless pump, moving blood from the atrium to the ventricle through the AVC during diastole, and from the ventricle to the vascular system during systole. On average, the flow is unidirectional between the atrium and the ventricle, but is oscillatory, and at times reversing in the AVC (Scherz et al., 2008; Vermot et al., 2009). Video recordings taken at a series of time points between 33 hpf and 48 hpf show this flow profile in the beating hearts of *Tg(gata1:dsred;fli:kaede)* embryos, in which RBCs and endocardial wall are labeled (Fig. S1A-F). These show that transiently reversing flows are almost continuous in the AVC between 36 hpf and 48 hpf. This is clearly visible when RBCs are advected from the ventricle to the atrium through the AVC in between the ventricular and atrial contractions (Fig. S1A-F). Similar results were previously discussed for *gata1* morphants (Vermot et al., 2009; Heckel et al., 2015). Thus, AVC development has a remarkable distinction from microcirculation morphogenesis, where endothelial cells tend to move away from oscillatory flows and upstream toward high shear stress regions with well-defined flow directionality in the vascular network (Kochhan et al., 2013; Franco et al., 2015; Kwon et al., 2016). Such cell behavior seems incompatible with the observed EdCs converging into the AVC, which suggests that the mechanical cue underlying EdCs behavior is more likely to be associated with the shear stress pattern generated by the oscillatory flow and its local reversals rather than that of the mean flow direction.

### Modeling hemodynamic wall shear stress and RBC contribution

To understand the shear stress patterns at work in the beating heart, we developed a model for blood flow in a simplified beating heart tube. It includes explicit RBCs that are expected to contribute to the shear stress pattern in space and time at the scales of the embryonic heart (Freund and Vermot, 2014; Fung, 1993; Boselli et al., 2015). The geometry is two-dimensional and includes key features of this flow: moving wall and flexible cells, with each RBC modeled as an elastic shell that can deform in response to shear flows and is fully coupled with the flow mechanics (see Materials and Methods). This simple RBC model is sufficient to reproduce key features of confined RBC channel flows, including the near-wall cell-free layers (Freund, 2007), rheological behaviors (Bryngelson and Freund, 2016a) and stability characteristics (Bryngelson and Freund, 2016b). The overall heart is represented as a linear tube with walls that move harmonically with amplitude and phase at each location based on the live imaging data. Our computational approach exploits the low Reynolds number of the zebrafish heart, ∼0.1, which means that inertia is negligible (Santhanakrishnan and Miller, 2011). Despite its obvious simplifications (see Materials and Methods), our model reproduces the key *in vivo* behavior: It is a valveless pump that forces an on-average unidirectional blood flow from the atrium inflow to the ventricle outflow. The flow is particularly unsteady, combining both pulsatile and oscillatory flow, matching that observed during AVC maturation. There are also reversals, with RBCs flowing backwards and forwards through the AVC (Fig. S1G-I, Movie 1). This model therefore recapitulates the main features of the embryonic heart and allows us to characterize the complex wall shear stress patterns arising under such flow conditions.

### Shear stress consists of periodic and nonperiodic components

**Fig. 3.**
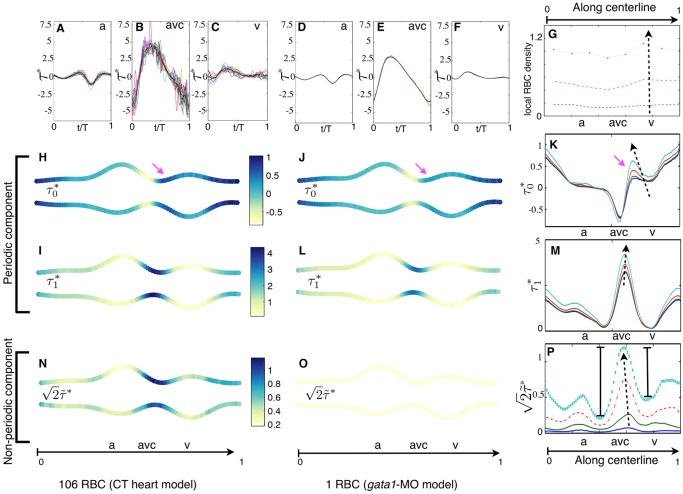
**Shear stress pattern during tissue convergence.** (A-F) Wall shear stress *τ* as a function of time at a point on (A,D) the atrial side (a), (B,E) the center (avc) and (C,F) the ventricular side (v) of the AVC. Different colors correspond to different heart cycles. Their phase-average (black line) is the periodic-component 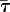 of *τ*. (H-K) Time average *τ*_0_ and (I-M) fundamental harmonic amplitude *τ*_1_ of the periodic component 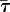 along the heart wall. (N-P) Nonperiodic oscillation 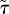 of the shear stress (scaled by 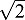 for consistency). A-C,H,I,N and D-F,J,L,O correspond to simulations with 106 and 1 RBCs (red blood cells), respectively. (G) RBC concentration along the heart. The results in K, M and P are for the upper wall and for different numbers of RBCs: *n*_*p*_=1, 17, 54,106 (dashed arrows point to larger values of *n*_*p*_). Pink arrows in H-K point out the uneven contribution of *n*_*p*_ to *τ*_0_. Asterisks denote results that are normalized by the space average of 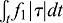.

To gain insight into the physical stimuli generated by the flow EdCs experience in the developing heart, we undertook a comprehensive analysis of the shear stress obtained with our simulations. The shear stress evaluated at the wall, which corresponds to the shear stress EdCs experience, *τ*(*t*), is a particularly unsteady signal because the endocardial walls are extremely dynamic in the embryonic heart. The wall shear stress signal is seen as the combination of two periodic components and one nonperiodic component: a constant signal, harmonic signals and nonperiodic oscillatory signals, respectively. The constant signal is the time average of the shear stress, *τ*_0_. The harmonic signals are sine functions and the frequency of each harmonic is a multiple of the heart beat frequency, which is the fundamental frequency of the system, *f*_1_. The amplitude of each harmonic signal can be computed via a standard Fourier analysis of the phase average of the shear stress signal over multiple heart beats. The average shear stress plus the harmonic oscillations represent the periodic component of the shear stress, 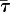. The nonperiodic component, 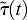, is associated with the chaotic dynamics of the RBCs (Fig. 3A-F), which is different at each heart beat, and is quantified as the root mean square (rtm) of the deviation of the shear stress signal from its periodic component: 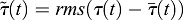 (Table S1).

To simplify our analysis, we considered these components separately. We first considered the periodic components of the wall shear stress. The time-average shear stress *τ*_0_ is largest on the boundaries of the AVC. It is positive (atrium to ventricle direction) at the ventricular side and negative (ventricle to atrium direction) at the atrial side of the AVC (Fig. 3H-K), which means that it is not linked to the atrium-to-ventricle mean direction of blood flow. We next considered the fundamental harmonic signal *τ*_1_ (oscillating with the heart beat frequency *f*_1_) and found that it makes the largest contribution to the wall shear stress in the AVC (Fig. 3I-M). Its maximum is at the center of the AVC. This confirms previous computations with a simpler model without RBCs (Heckel et al., 2015; Boselli and Vermot, 2016). Higher frequency harmonics (for example *τ*_2_, *τ*_3_,…, oscillating with frequency 2*f*_1_, 3*f*_1_,…) have a similar profile though lower amplitude (Fig. S2). Finally, we considered the nonperiodic component (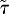) and found it is maximum at the AVC region and with peak values comparable to those of the time average *τ*_0_ (Fig. 3N,P), resembling the profile of *τ*_1_. These results suggest that the pattern of each shear stress component represents a physical landmark cells could specifically sense in the AVC.

### Impact of RBCs on the pattern of the shear stress components

We next addressed the contribution of RBCs on the components of the shear stress distribution in order to better characterize the *gata1* morphants devoid of RBCs, and to assess the robustness of our results with respect to the stiffness and shape of the RBC model. The amplitude of the fundamental harmonic signal *τ*_1_ and the other harmonics increase almost linearly with the number of RBCs, *n*_*rbc*_, and therefore preferentially in the AVC. By contrast, the pattern of the average shear stress *τ*_0_ is more sensitive to *n*_*rbc*_, and increases unevenly with *n*_*rbc*_ at the ventricular side of the AVC (Fig. 3H-K). This effect depends on the uneven distribution of the RBCs and the formation of RBC-depleted regions (Fig. S1K; Fig. 3G). In our simple model, depleted regions can be observed, for example, close to the upper (as shown) atrial wall at the AVC inflow (Fig. S1K), where the wall shear stress increases slower with the number of RBCs (Fig. 3K). Live imaging of *Tg(gata1:dsred)* also shows RBC clustering and RBC-depleted regions (Fig. S1L,M), suggesting that a nonintuitive contribution of RBCs to wall shear stress patterning must be expected in the beating heart as well. However, the pattern of both *τ*_0_ and *τ*_1_, including the direction of the former, do not change qualitatively, demonstrating that the results for the periodic components hold independently of the number of RBCs used in the simulations.

Not surprisingly, we found that the nonperiodic component 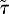 goes to zero for *n*_*rbc*_=0 (Fig. 3O). As for *τ*_0_, RBC clustering alters the pattern of 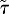 such that nonperiodic oscillations are less important in RBC-depleted regions (Fig. 3P).

Finally, while the stiffness and shape of the RBCs impact wall shear stress, with stiffer RBCs leading to stronger values of all the shear stress components (Fig. S3), the patterns of each component of the wall shear stress remain qualitatively the same. Thus, our results are independent of the physical parameters describing the RBC model.

### The computed periodic components of wall shear stress predict tissue convergence orientation

We next dissected the biological importance of each shear stress component as a mechanical cue for tissue convergence in the AVC by comparing the results obtained experimentally with those from our simulations. As discussed, tissue convergence in the AVC is also observed in the hearts of *gata1* morphants (Fig. 2F), where the nonperiodic contribution of the RBCs (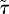) is negligible, such that 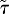 does not appear to be a necessary mechanical cue for this specific morphogenetic event.

By contrast, the major features of the periodic components of the shears stress are conserved in the wild types and *gata1* morphants and are potential cues for tissue convergence. We thus conclude that the periodic components are the best predictor of EdC convergence in the AVC observed *in vivo*, but not flow direction or the nonperiodic component.

### Mechanistic model and conclusion

**Fig. 4.**
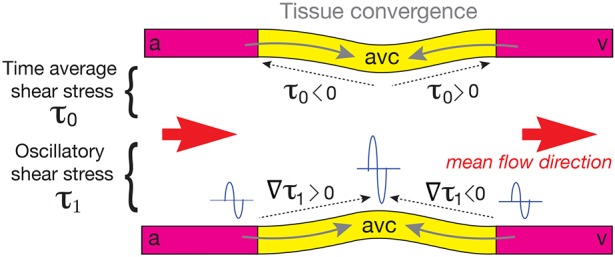
**Schematic** **of the AVC illustrating the relationship between the directions of the converging movements of EdCs, mean shear stress, gradient of the amplitude of the fundamental harmonic** **and average flow.** Gray arrows indicate the directions of the converging movements of the EdCs, dotted arrows (upper wall) indicate the direction of the mean shear stress *τ*_0_, dotted arrows (lower wall) indicate the direction of the gradient of the amplitude of the fundamental harmonic 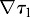, and orange arrows indicate the average flow direction. Tissue moves toward higher values of the amplitude of the fundamental harmonic *τ*_1_ (illustrated with sine waves), which is in the same direction as its gradient 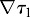 and against the direction of the time average shear stress *τ*_0_. This does not match with the direction of the average blood flow (orange arrows), which is from atrium (a) to ventricle (v) along the heart. The center of the AVC is in yellow and the atrial and ventricular sides are in magenta. This configuration is a direct consequence of the typical reversing flow observed at 36-48 hpf in the AVC.

We considered the early morphogenetic events between 36 hpf to 48 hpf that ultimately lead to AVC maturation and valve formation. Using live imaging and three-dimensional image analysis, we showed that EdCs converge into the AVC and this process depends upon mechanical forces. Mathematical modeling allowed us to analyze the shear stress patterns associated with the reversing, oscillatory flow in the AVC. Though previous studies have illuminated the fluid dynamics of the developing heart (Jamison et al., 2013; Lee et al., 2013; Heckel et al., 2015; Miller, 2011; Taber et al., 2007; Midgett et al., 2015; Boselli and Vermot, 2016), this model allows us to account for the cellular character of blood in the heart at these stages. The highly unsteady, oscillatory, reversing flow, together with the dynamic boundaries of the endocardial walls give the flow and shear stress patterns in the AVC a complexity exceeding that of the rest of the circulation (Anton et al., 2013). The direction of the time average shear stress, *τ*_0_, and the gradient of the fundamental harmonics amplitude, 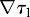, does not match with the mean direction of blood flow (Fig. 4). Instead, they point away from and toward the AVC center, respectively (Fig. 4). Thus EdCs move in the direction of higher oscillation amplitudes of the phase average wall shear stress and opposite to the direction of the time average *τ*_0_, thus converging in the AVC (Fig. 4).

This study leaves us with several unresolved questions. First, the cellular mechanisms leading to tissue convergence in the AVC remain an open issue, but we speculate that cell intercalation (Tada and Heisenberg, 2012), cell proliferation and/or cell migration (Eder et al., 2016; LeGoff and Lecuit, 2016) are involved. Second, future work using realistic three-dimensional geometries will be necessary to study potential azimuthal patterns involved in nontrivial force generation. Third, beside shear stress, additional mechanical stimuli might be generated at the cellular scale, such as the strain associated with cell deformation and myocardial contractility. Thus, it will be important to assess the respective impact of myocardial contractility, the strain and the shear stress exerted on the endocardial cells behaviors. It is to note that our model does not suggest any obvious pattern of normal stress (perpendicular to the endocardium) that could establish a convergence zone in the AVC. However, we do not have a constitutive model of the heart wall and its coupling with the vascular system and thus we cannot be conclusive on the role of tissue strain and normal forces.

Overall, our results suggest that the reversing, oscillatory flow in the AVC leads to particular shear stress patterns that can establish a convergence zone in the AVC though the average blood flow is unidirectional (Fig. 4). Thus, shear stress patterns rather than flow directionality are potential determinants of the flow dependent morphogenetic events leading to valve development.

## MATERIALS AND METHODS

### Zebrafish husbandry, embryo treatments and morpholinos

Animal experiments were approved by the Animal Experimentation Committee of the Institutional Review Board of the IGBMC. Zebrafish lines used in this study were *Tg(fli1a:gal4FUBS; UAS:kaede)* (Herwig et al., 2011), *silent heart* (*sih*) (Sehnert et al., 2002), and *Tg(gata1:dsRed)* (Traver et al., 2003). For specific experiments, a morpholino for *gata1* (*gata1* MO) was injected into the yolk at the one-cell stage as described previously (Galloway et al., 2005; Vermot et al., 2009). All animals were incubated at 28.5°C for 5 h before treatment with 1-phenyl-2-thiourea (PTU) (Sigma-Aldrich) to prevent pigment formation.

### Photoconversion and *in vivo* imaging

Photoconversion experiments and live imaging were performed using the FRAP module of a confocal microscope (Steed et al., 2016b). *Tg(fli1a:gal4FUBS; UAS:kaede)* hearts were exposed to 405 nm light to convert the Kaede protein to its red form. A standard *z*-stack of the photoconverted heart was then acquired at both 36 hpf and 48 hpf. See Supplementary Materials and Methods for details.

### Three-dimensional analysis and statistical analysis

The three-dimensional analysis of the photoconverted cells was performed using a newly developed approach implemented in MATLAB. The AVC region was segmented, fitted with a parametric surface, and unfolded for two-dimensional visualization and quantification of the shortening factor (*L*_48_−*L*_36_)/*L*_36_, with *L*_36_ and *L*_48_ the length of the nonphotoconverted AVC region at 36 hpf and 48 hpf, respectively. See supplementary Materials and Methods for details. We repeated the experiment twice, and analyzed *n*=5 controls, *n*=4 *sih* mutants and *n*=9 *gata1* morphants. The statistical significance of the differences between the mean values calculated for control and perturbed flow conditions was determined by unpaired Student's *t*-tests. See supplementary Materials and Methods for details.

### Computational modeling

Fluid dynamic simulations were based on a new research code based on efficient boundary integral methods (Freund, 2007) for the solution of Stokes flow equations. This exploits the low Reynolds number, Re, of the zebrafish heart (Re<1), where inertia is almost negligible and the linearization underling the Stokes flow equations is appropriate. See supplementary Materials and Methods for details.

## Supplementary Material

Supplementary information

Supplementary information
