# Supplementary material for: Anisotropic shear stress patterns predict the orientation of convergent tissue movements in the embryonic heart
Source: Development. 2017 Dec 1;144(23):4322–7. doi: 10.1242/dev.152124 (PMC5769631; doi:10.1242/dev.152124)
Supplement: Supplementary information [file develop-144-152124-s1.pdf]

Supplementary Material

Materials and Methods

Materials and Experimental Methods

Photoconversion and in vivo imaging Photoconversion was performed using the FRAP module of a confocal microscope (TCS SP8, Leica microsystems) equipped with Leica HCX IRAPO L, x25, NA0.95 water immersion objective, as described previously (Steed et al., 2016).

Tg(fli1a:Gal4FF; UAS:Kaede) embryos were mounted in 0.7% low melting-point agarose (Sigma Aldrich) supplemented with 50 mM BDM to inhibit heart contraction at about 36hpf. Regions in the ventricle and the atrium were exposed to 405 nm light by applying 35 bleach pulses (35 ms each; 25% laser power). This converted the kaede protein to its red form. A standard z-stack of the photoconverted heart at 36hpf was then acquired, with 2 μm between the z-sections.

Embryos were then carefully dissected from the agarose, placed in fish water for 5-10 min until heart contraction resumed and then put at 28.5 °C to develop individually under standard conditions until 48hpf. A second z-stack was then acquired at 48hpf under the same conditions as for the 36hpf acquisitions.

In order to analyze tissue movement under perturbed flow conditions, the same experiment was repeated for the *Tg(fli1a:Gal4FF; UAS:Kaede)* injected with *gata1* MO, and for crosses of *sih* and *Tg(fli1a:Gal4FF; UAS:Kaede)*.

Live imaging of the beating heart was performed using light sheet microscopy (TCS SP8 DLS, Leica Microsystem) for the flow analysis between 36 and 48 hpf (Fig. S1) and confocal microscopy (TCS sp8, Leica microsystems) for the AVC shortening analysis. z-stacks of the beating hearts were synchronized post acquisition as previously described (Liebling et al., 2006).

Development • Supplementary information

AVC unfolding, quantification of the AVC shortening, and statistical analysis The three-dimensional movement of the photoconverted cells within each heart was analyzed using a newly developed approach.

The following steps are implemented in Matlab scripts.

- Step 1: Points on the endocardium are identified by intensity thresholding of the acquired images and plotted in three dimensions. Points on the atrium and the ventricle are erased interactively using the erase command of Matlab to isolate points representing the narrowest and most straight part of the AVC. These points are selected and fitted with a cylinder as described previously (McMahon et al., 2008). A reference system can then be introduced with the axis of the cylinder as z-axis and the center of mass of the AVC points as origin. The endocardial points are reoriented such that the points on the ventricle have a positive z- (axial) position. The heart points are then rotated around the z-axis with this strategy: The AVC points are projected on the x-y plane and fitted with an ellipse. The endocardial points are then rotated around the z-axis to bring the major ellipse axis parallel to the y-axis and such that the internal side of the AVC (with respect to the embryo) points has positive y-values. At this point, the angular position of cells in the AVC is defined consistently for all the hearts.

- Step 2: The three-dimensional dataset is cut by planes obtained by rotating the half-plane {y=0, x>0} in the z-direction. The intensity of the neighboring pixels are projected on these planes and the endothelium traced with a line of points ordered with increasing arc-length from the atrium to the ventricular side of the AVC. These points are then interpolated by a three dimensional spline (spline toolbox, matlab). Each point on the resulting surface have a well defined angular position and the tissue length between two points at the same angular position is computed as the archlenght on the surface between the two

Development • Supplementary information

points. This is different than the length of a linear segment between two points because the endocardium is curved.

- Step 3: The intensity of the neighboring pixels are projected on the parametric surface. This gives a two-dimensional image with each column corresponding to the line on the surface at a given angle. This is equivalent to unfold the AVC into a two-dimensional image.

- Step 4: The unfolded AVC can then be segmented in two-dimensions with standard tools to find the edges of the non-photoconverted AVC and the AVC length L as a function of the angular position.

The same analysis was repeated at 36 and 48 hpf for each heart considered, and the AVC shrinking factor was computed as $(L_{48} - L_{36})/L_{36}$ as a function of the angular position and averaged over the inferior, superior, internal and external regions of the AVC (Fig. 2), which are defined consistently based on the orientation of the elliptic cross-section of the AVC and its orientation within the embryo.

The statistical significance of the differences between the mean values calculated for control and perturbed flow conditions was determined by unpaired Student's *t*-tests and computed with the `ttest2` function of the Matlab Statistical Toolbox, without assuming equal variances. Shown standard deviations were computed as corrected sample standard deviations using the `std` Matlab function.

Computational Methods

Heart dynamics and anatomy We exploit the viscous regime of the zebrafish heart, where the Reynolds number is reported to vary from 0.017 at 26hpf to 0.342 at 4.5 days post fertilization (Santhanakrishnan and Miller, 2011). Therefore we neglect inertia effects, though they

Development • Supplementary information

might alter details at 4.5dpf, as well as the overall curvature of the heart which will not alter flow substantively at so low of Reynolds numbers. In two dimensions, the dynamics of a point on the wall is simply defined as: $y(x,t) = d_0 + A\sin(t/f_1 2\pi + \theta_1)$ for both the upper and lower walls, where we truncate the Fourier expansion of $d(t)$ to the heart beat frequency f_1. The time average radius d_0 and the oscillation amplitude A and the beating frequency f_1 were previously extracted from live imaging of 48hpf zebrafish hearts as described previously (Boselli and Vermot, 2016). Previous works showed how two-dimensional models can well predict typical shear stress in the heart of zebrafish embryos (Lee et al., 2013; Boselli and Vermot, 2016), which is the focus of this work. Therefore, despite the fact that an accurate description of some hemodynamic cues, including pressure, would require a three-dimensional model of the heart and of the hydrodynamic load represented by the vascular system, we will simplify the system and limit our analysis to two dimensions.

RBC model The membrane of each red blood cell (RBC) is modelled as an elastic shell of tension modulus T and bending modulus M. The interior of the cell is molded as a fluid with the same viscosity as the plasma surrounding the cell. The flow of the plasma and of the cytosol are treated as Stokesian such that the flow velocity u_i can be expressed by the boundary integral

$$u_i(\mathbf{x},\mathbf{t}) = \frac{1}{4\pi\mu}\int_\Omega S_{ij}(\mathbf{y}-\mathbf{x})\,\Delta\sigma_j(\mathbf{y})\,ds(\mathbf{y}), \tag{1}$$

where S_{ij} is the Stokeslet tensor, s is the arc-length position of a point $\mathbf{x}(s)$ on the membrane, and $\Delta\sigma_j$ is the traction of the membrane on the fluid. For the cell membrane, the traction is given by the relation:

$$\Delta\sigma_i = \frac{\partial\tau}{\partial s}t_i + \frac{\partial}{\partial s}\left(\frac{\partial b}{\partial s}n_i\right), \tag{2}$$

with τ the membrane tension,

$$\tau = T\left(\frac{ds}{ds_0} - 1\right), \tag{3}$$

Development • Supplementary information

and b the bending moment,

$$b = M\left(C(s) - C_0\right), \tag{4}$$

with C the curvature and C_0 a constant initial curvature that cancels out in (2). The coordinate s_0 in Eqations (3) and (4) is the referential arch-length position of a point on the cell membrane in an hypothetic stress-free configuration. In practice, the stress-free shape of the RBCs does not need to be defined. It is sufficient to set the perimeter l_0 of the stress-free membrane and the area $r_0^2\pi$ of each of our two-dimensional RBCs such that $l_0 \neq 2\pi r_0$. Starting from an arbitrary initial configuration (here a sphere of perimeter $2\pi r_0$), the RBC will assume a configuration that minimize the elastic energy of the membrane. The time required by this process scales like the relaxation time of the red blood cell $\tau_{rlx} = r_0\mu/T$, and its ratio with the convective time scale $\tau_U = r_0/U$ gives a non dimensional index of the elasticity of the red blood cells (Freund, 2007). The main simulations were set-up such that $\tau_{rlx}/\tau_U = 0.16$, $r_0^2 T/M = 12.5$ and $l_0/(2\pi r_0) = 1.6\pi$. Simulations presented in Fig. S3, where repeated for $l_0/(2\pi r_0) = 1.2\pi$, $\tau_{rlx}/\tau_U = 0.1$, and $\tau_{rlx}/\tau_U = 0.32$, which, in the order, correspond to rounder, stiffer and softer red blood cells. The research code is available upon request.

Wall model The heart walls dynamics is obtained by imposing the wall traction

$$\Delta\sigma_i^w = -k_w(x_i - x_i^w). \tag{5}$$

which is equivalent to link each point on the wall to their desired location $\mathbf{x}^w$ by a virtual spring of stiffness k_w, which was kept constant and such that $k_w r_0^2/T = 1.5$ in the main simulations.

The flow velocity due to the wall traction can be expressed by the same boundary internal formulation (1) used for the cell membrane, but with $\Delta\sigma_i^w$ instead of $\Delta\sigma_i^m$. As for the RBCs, the number of points necessary to describe the shape of the wall is smaller than that required

Development • Supplementary information

to compute the integrals along the wall. Only these control points are carried on during time integration. The extra collocation points for the space integrals are then obtained by Fourier interpolation, which exploits the periodicity of the boundary.

Time integration A point $\mathbf{x} = \{x_i\}$ on the cell membrane or on the wall moves then according to

$$\frac{\partial x_i}{\partial t} = u_i(\mathbf{x}, t), \tag{6}$$

where u_i now is the sum of the contribution of all the cell membranes and the walls in the model system. Time integration is performed by a second order Runge-Kutta scheme. The time step dt was set such that $dt * f_1 = 5 \cdot 10^{-5}$.

Numerical discretization The boundary integral (1) is computed by a quadrature around the periodic membranes (cells and walls). The quadrature can be computed on a uniform grid by discretizing the membrane with N_a points uniformly spaced in s_0 and by expressing the Cartesian $\mathbf{x}(\mathbf{s_0})$ and the archlength $s(s_0)$ coordinates as a function of s_0:

$$u_i^m = \frac{1}{4\pi\mu} \sum_{n=1}^{N_a} S_{ij}(\mathbf{x}^n - \mathbf{x}^m)\, \Delta\sigma_j^n \, \frac{ds}{ds_0}|^n \Delta s_0 \tag{7}$$

$$\text{for} \quad m = 1 \ldots N;$$

where N_a is the number of points on the cell membrane and wall used for the quadrature and $N = N^m + N^w + N^v$ is the number of evaluation points, which is the sum of N^m collocation points used to represent the cell membrane, N^w wall points, and N^v points where to evaluate u_i for post processing or simply for visualization.

The numerical discretization, including the PME method employed to speed up the evaluation of (7), is described in details in (Freund, 2007). Starting from the results in (Freund, 2007), the collocation points on the membrane of each RBCs was set to 128, and to 800 on the wall.

Development • Supplementary information

Computation of the shear stress The integral boundary methods extend the flow domain over the whole space such that flow is also computed outside the actual domain of interest. Therefore, the wall shear stress at the endocardial interface is computed as $\tau_w = \sigma_i^w t_i^w + \tau_{out}$, where t_i^w is the unit tangent vector to the wall and τ_{out} is the wall shear stress due to the flow outside the heart model. This is computed as $\tau_{out} = (u_i|_d t_i^w - u_i|_w t_i^w)/d$ where d is chosen to minimize $\sigma_i^w t_i^w - \tau_{out} - \tau_{in}$ without RBCs. τ_{in} is not computed directly to avoid the finite difference kernel to overlap with an RBC passing very close to the wall.The research code is available upon request.

References and Notes

Boselli, F. and Vermot, J. (2016). Live imaging and modeling for shear stress quantification in the embryonic zebrafish heart. Methods *94*, 129–134.

Freund, J. B. (2007). Leukocyte margination in a model microvessel. Phys. Fluids *19*, 023301.

Lee, J., Moghadam, M. E., Kung, E., Cao, H., Beebe, T., Miller, Y., Roman, B. L., Lien, C.-L., Chi, N. C., Marsden, A. L. and Hsiai, T. K. (2013). Moving Domain Computational Fluid Dynamics to Interface with an Embryonic Model of Cardiac Morphogenesis. PLoS ONE *8*, e72924.

Liebling, M., Vermot, J., Forouhar, A. S., Gharib, M., Dickinson, M. E. and Fraser, S. E. (2006). Nonuniform temporal alignment of slice sequences for four-dimensional imaging of cyclically deforming embryonic structures. In 3rd IEEE International Symposium on Biomedical Imaging: Nano to Macro, 2006. pp. 1156–1159,.

McMahon, A., Supatto, W., Fraser, S. E. and Stathopoulos, A. (2008). Dynamic analyses of Drosophila gastrulation provide insights into collective cell migration. Science *322*, 1546–50.

Santhanakrishnan, A. and Miller, L. A. (2011). Fluid Dynamics of Heart Development. Cell Biochemistry and Biophysics *61*, 1–22.

Steed, E., Faggianelli, N., Roth, S., Ramspacher, C., Concordet, J.-P. and Vermot, J. (2016). klf2a couples mechanotransduction and zebrafish valve morphogenesis through fibronectin synthesis. Nat Commun *7*, 11646.

Development • Supplementary information

Supplementary figures and movies

Supplements include one movie: Movie 1; there supplementary figures: Fig. S1, S2, 3; and Table S1.

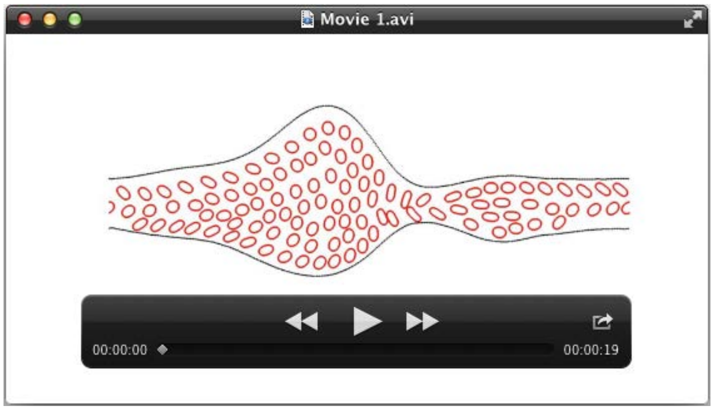

Movie 1: Related to Fig. S1G-I and 3. This movie illustrates the dynamics of 106 red blood cells (red) and of the walls (black) in the proposed computational model of the embryonic heart.

symbol	definition
τ	wall shear stress
$\bar{\tau}$	phase average of τ
τ_0	time average of τ
τ_1	fundamental harmonic component of τ, oscillating with the same frequency as the heart f_1
τ_n	harmonic component of τ oscillating at frequency f_n
$\tilde{\tau}$	root mean square (rms) amplitude of non periodic oscillations associated to the chaotic flow of red blood cells

Table S1: Brief definition of the symbols used in the analysis of the wall shear stress.

Development • Supplementary information

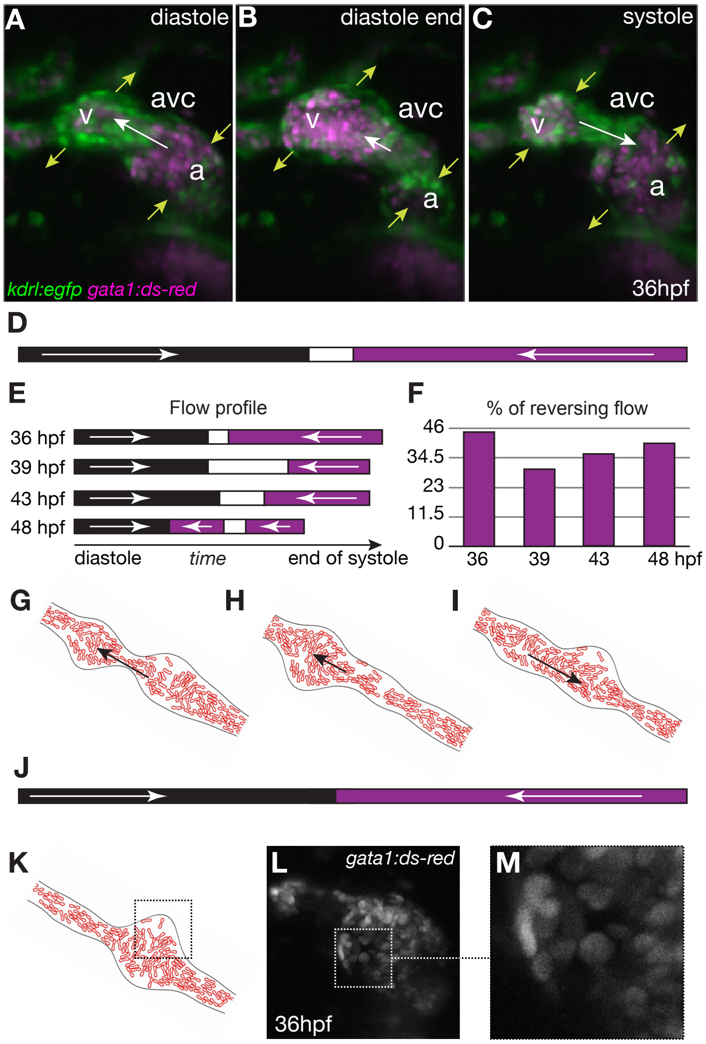

Figure S1: Reversing, oscillatory flow in the AVC between 36 and 48 hpf. A-C) Maximum projection of the three-dimensional reconstruction of a 36 hpf *Tg(fli:kaede;gata1:dsRed)* zebrafish heart at three different instants of the heart beat. The dsred labelled red blood cells are shown in magenta, while endocardial cells are shown in green. D,E) Flow direction in the AVC at D) 36hpf and E) between 36-48 hpf: magenta, flow moves from atrium to ventricle; black, flow is from ventricle to atrium; white, no flow or not visible. F) Average percentage of reversing flow per heart beat. G-I) Computational model for RBC flows between 36-48 hpf at three different instants of the heart beat (cf. Movie 1). J) Flow direction in the AVC model: solid arrows point in the direction of the flow. Dashed squares point out the uneven-time dependent distribution of RBCs in K) the model, and L,M) in vivo.

Development • Supplementary information

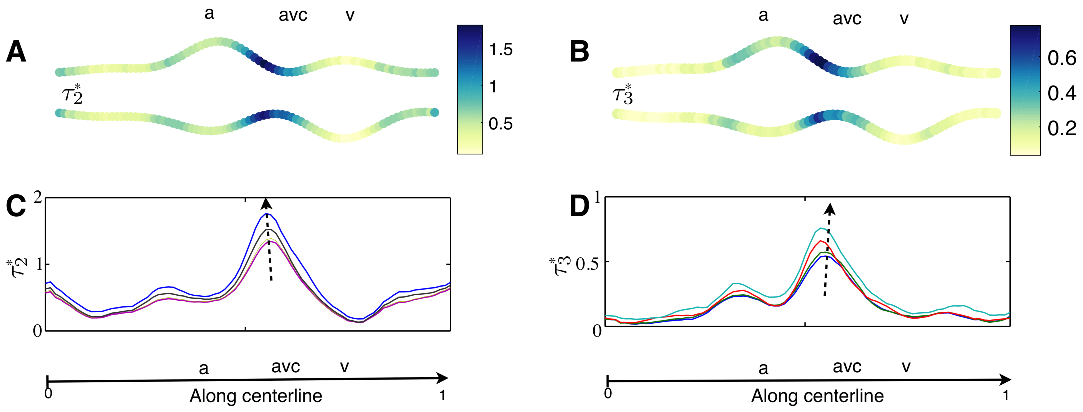

Figure S2: Shear stress pattern during tissue convergence (higher harmonics) in complement to Fig. 3. A,C) Second (τ_2) and B,D) third (τ_3) harmonics. The results in A and B are for 106 red blood cells. The results on C and D are for the upper wall and for different numbers of red blood cells n_p =1, 17, 54,106 (arrows point to larger values of n_p). The superscript * denotes that results are normalized by the space average of $\int_t f_1|\tau|dt$. a: atrium; avc: atrioventricular canal; v: ventricle.

Development • Supplementary information

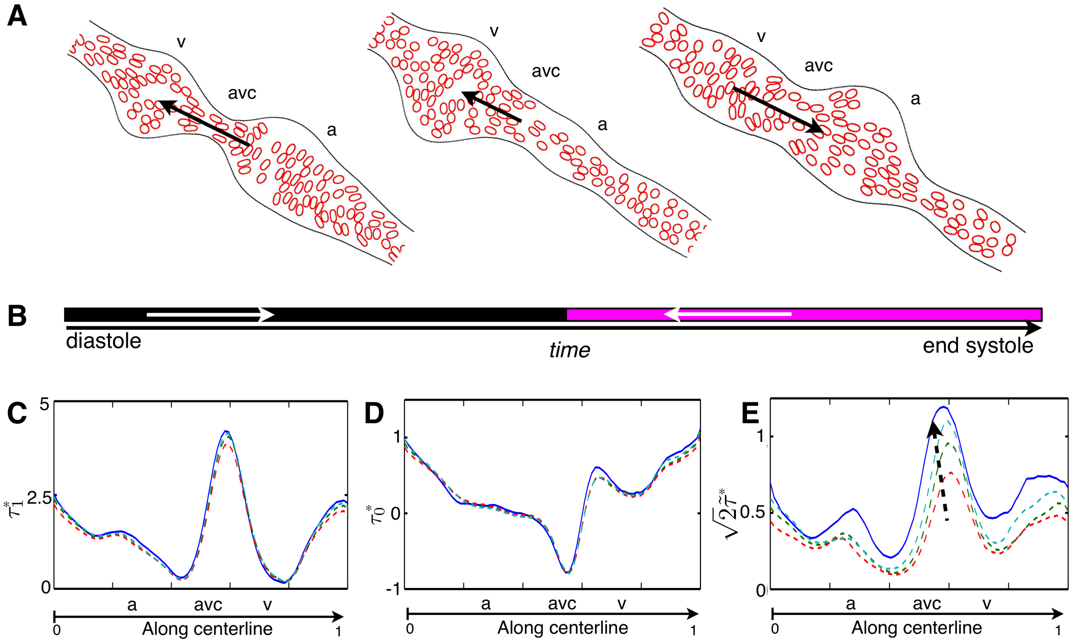

Figure S3: Model sensitivity to red blood cell (RBC) shape and stiffness. Related to Fig. 3. A) Computational model for RBC flows at three different instants of the heart beat for round elastic red blood cells (cf. Fig. S1G-I). B) Flow direction in the AVC model: magenta, flow is from atrium to ventricle; black, flow is from ventricle to atrium; white, no flow or not visible. Solid arrows point in the direction of the flow. C) Fundamental harmonic τ_1, D) time average τ_0, and E) non-periodic oscillation $\tilde{\tau}$ of the shear stress. C-E) The blue solid line corresponds to the RBC shape of the test case of Fig. S1 and 3, n_p =106, and $\tau_{rlx}/\tau_U = 0.16$. The three dashed lines correspond to the rounder RBC shape shown in A and are obtained using the same, stiffer ($\tau_{rlx}/\tau_U = 0.1$), and softer ($\tau_{rlx}/\tau_U = 0.32$) RBC membranes (dashed arrow points to larger value of stiffness). The superscript * denotes that results are normalized by the space average of $\int_t f_1|\tau|dt$. a: atrium; avc: atrioventricular canal; v: ventricle.

Development • Supplementary information
